# Esophageal perforation: diagnostic work-up and clinical decision-making in the first 24 hours

**DOI:** 10.1186/1757-7241-19-66

**Published:** 2011-10-30

**Authors:** Jon Arne Søreide, Asgaut Viste

**Affiliations:** 1 Department of Gastroenterologic Surgery, Stavanger University Hospital, N-4068 Stavanger, Norway; 2 Department of Gastroenterologic Surgery, Haukeland University Hospital, N-5021 Bergen, Norway; 3 Department of Surgical Science, University of Bergen, N-5021 Bergen, Norway

**Keywords:** Esophagus, perforation, early diagnosis, surgery, non-surgical management, endoscopy

## Abstract

Esophageal perforation is a rare and potentially life-threatening condition. Early clinical suspicion and imaging is important for case management to achieve a good outcome. However, recent studies continue to report high morbidity and mortality greater than 20% from esophageal perforation. At least half of the perforations are iatrogenic, mostly related to endoscopic instrumentation used in the upper gastrointestinal tract, while about a third are spontaneous perforations. Surgical treatment remains an important option for many patients, but a non-operative approach, with or without use of an endoscopic stent or placement of internal or external drains, should be considered when the clinical situation allows for a less invasive approach. The rarity of this emergency makes it difficult for a physician to obtain extensive individual clinical experience; it is also challenging to obtain firm scientific evidence that informs patient management and clinical decision-making. Improved attention to non-specific symptoms and signs and early diagnosis based on imaging may translate into better outcomes for this group of patients, many of whom are elderly with significant comorbidity.

## Introduction

Esophageal perforation is a well-characterized and potentially life-threatening clinical situation [1-3]. Several factors, including the difficulty of accessing the esophagus, the lack of a strong serosal layer, the unusual blood supply of the organ and the proximity of vital structures, all contribute to this condition's high morbidity and to a mortality rate of at least 20% [4-6]. In addition, the diversity of clinical symptoms and signs combined with a lack of individual experience regarding this particular condition may impede rapid identification of this potentially hazardous situation. Accordingly, delayed diagnostic work-up may hinder timely and appropriate treatment with a negative effect on patient outcome [7].

The scientific evidence that guides management of esophageal perforation is based mainly on retrospective studies at single institutions as well as on a few nationwide studies [1,4-6,8-10]. Randomized studies are non-existent. Nevertheless, attention should be paid to early diagnosis and immediate treatment to save lives and to decrease morbidity and long-term sequelae.

In this review, we focus on the clinical aspects of esophageal perforation that are most helpful for early suspicion and that should prompt appropriate diagnostic tasks. We also highlight factors with particular clinical importance for informed decision-making during the first 24 hours of treatment in-hospital.

### Incidence and demographics

The low incidence of esophageal perforation is supported by reports from institutional series that are often based on consecutive patients diagnosed over a period of decades [9,11-13]. However, referral bias and publication bias may obscure accurate incidence statistics. In a recent population-based study in Iceland, the age-standard incidence was 3.1/1 000 000/year [6]. Nevertheless, the true incidence of esophageal perforation worldwide is not clear [14]. Most patients are in their sixties, and esophageal perforation is slightly more common in males [5].

### Causes

Esophagus rupture is usually iatrogenic [6,8,15], the result of endoscopic procedures [16] such as esophageal dilatation for strictures and for achalasia in particular [17]. It can also result from surgery on tissue that is in close proximity to the esophagus [18-20]. In about 15% of the cases, there is spontaneous rupture with no known pre-existing pathology of the esophagus. This is mostly related to intense vomiting or severe retching, which probably causes an increase in intra-abdominal pressure. This clinico-pathologic entity, first described by Hermann Boerhave in 1724, was originally called Boerhave syndrome [21]. It is likely that very rare esophageal ruptures related to weight lifting, parturition, status epilepticus, defecation or to use of the Heimlich maneuver result from the same underlying mechanisms. Rare causes of perforation include external air-blast trauma [22] and blunt trauma [23].

Penetrating sharp injuries, i.e. external trauma, can damage the superficially located cervical esophagus as well as the thoracic portion of the esophagus. Although rare, gunshot wounds can cause tissue damage that can be easily missed during examination. Therefore, a high index of suspicion of esophageal perforation is warranted whenever there are penetrating injuries in this region [24].

In children, injuries to the esophagus are usually due to accidental ingestion of caustic liquids [25]. In contrast, the ingestion of caustic substances by adults is usually associated with suicidal intentions [26]. Cleaners, battery liquids and solutions used in industrial operations can generally be classified as acids or alkalis [27]. While acids, most of which have an unpleasant taste, produce coagulative tissue necrosis with a lower risk of penetration, alkalis tend to be more palatable and cause liquefactive necrosis that rapidly becomes transmural. The injuries and clinical consequences of ingestion of caustic substances depend on several factors, including the amount, viscosity and concentration of the agent, as well as on the duration of contact between the caustic agent and the esophageal mucosa.

### Symptoms and signs of esophageal perforation

While chest pain is regarded as the cardinal symptom of esophageal perforation and is present in more than 70% of patients with a full thickness perforation of the intrathoracic esophagus, other symptoms and signs are variable and nonspecific in many patients. Notably, a missed diagnosis that was first made at autopsy has been reported in 17% of cases [6]. The pain associated with esophageal perforation is usually acute and sudden in onset, with radiation to the back or to the left shoulder. In about 25% of the patients, this pain is followed by vomiting and shortness of breath. The triad of vomiting, chest pain and subcutaneous emphysema is known as the Mackler triad [28].

There is neck pain when the cervical esophagus is perforated, although systemic symptoms are less common. Dysphonia, hoarseness, cervical dysphagia and subcutaneous emphysema are encountered in various combinations in this group of patients. There is sometimes acute abdominal or epigastric pain in patients with perforation of the gastroesophageal junction. Notably, perforations rarely manifest with hematemesis or other signs of gastrointestinal bleeding, including melena.

Most patients are in significant distress upon physical examination. Tachycardia is common, with fever (> 38.5°C) as a later sign. Attention should be paid as to whether there is crepitus in the neck region or at the chest wall, as this is characteristic of subcutaneous emphysema. A systemic inflammatory response usually develops rapidly after perforation, generally within 24-48 hours, and overwhelming bacterial mediastinitis may cause cardiopulmonary collapse and multiple organ failure (MOF) with a fatal outcome within a short period of time. Thus, diagnostic work-up should be performed as soon as esophageal perforation is considered a tentative diagnosis based on symptoms, signs, the patient's recent history (e.g. use of medical instruments or interventions in the esophagus, episodes of acute vomiting, ingestion of foreign bodies or agents) and careful clinical examination.

### Imaging and work-up

**Figure 1:**
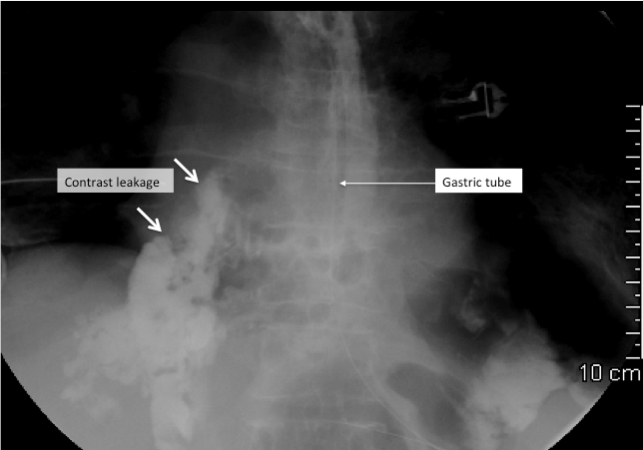
**Plain chest radiography with a water soluble contrast swallow, showing contrast leakage in a patient with spontaneous rupture of the esophagus**.

Diagnosis of an esophageal perforation relies on radiographic evidence. Specifically, indirect signs of esophageal injury can be seen on a posteroanterior and lateral *plain chest radiograph*. Such signs include pleural effusion, pneumomediastinum, subcutaneous emphysema, hydrothorax, pneumothorax and collapse of the lung. However, a *chest radiograph that uses a water-soluble contrast medium *(if the patient can swallow) will reveal a contrast leak in most cases of esophageal perforation (Figure 1) [29]. Water-soluble contrast should be used instead of barium contrast to prevent barium-related inflammation of the mediastinum if there is perforation. If the initial contrast-swallowing study is negative, imaging should be repeated after 4-6 hours if the clinical suspicion remains.

**Figure 2:**
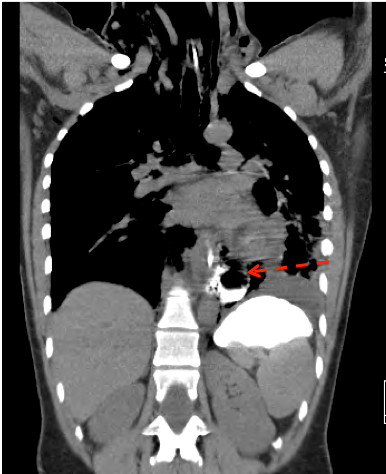
**Computer tomography (CT) with an oral contrast swallow, showing distal contrast leakage and gas bubbles in the mediastinum only few hours after pneumatic dilatation for achalasia**.

*Computer tomography (CT) *of the chest and upper abdomen with oral contrast can also show whether there is a leak [30](Figure 2). In addition, collection of air or fluid in the mediastinum, pleural effusions, pneumocardium and pneumoperitoneum are important diagnostic findings in these patients [31-33]. The site of perforation and the degree of containment may be easier to judge by CT than by plain chest X-ray. In critically ill patients and in those with external trauma or caustic injuries, a CT examination may provide additional information.

**Figure 3:**
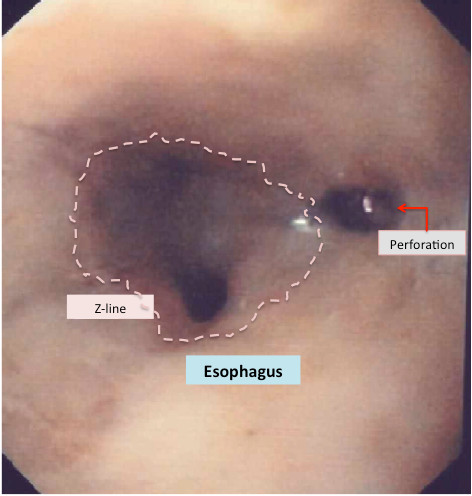
**Endoscopic view of a distal spontaneous perforation 24 hours after onset of clinical symptoms, according to the patient**. Endoscopic appearance, however, may suggest a time period exceeding at least 36-48 hours from onset of symptoms to endoscopic diagnosis.

The role of *upper endoscopy *in the early diagnostic work-up of patients with suspected esophageal perforation has been disputed [34]. In a patient with high suspicion of perforation with negative radiography, or when swallowing a contrast agent is impossible for technical reasons, flexible endoscopy should be considered. This widely available tool allows direct visualization of the entire esophagus and stomach and may also provide additional information about the acute onset of symptoms in patients without previous instrumentation used in the esophagus (Figure 3).

### Early management and decision-making

Patients with suspected esophageal perforation should be regarded as critically ill [21]. It is important to adopt an immediate and aggressive diagnostic approach to confirm the diagnosis and to identify related issues. Nil per mouth, intravenous fluids and appropriate pain treatment should be initiated. Broad-spectrum antibiotics should be given intravenously [1], and oxygen saturation should be monitored. Appropriate observation and management of these patients usually requires the resources of an intensive care unit along with careful and close surgical guidance and post-surgery surveillance.

While most surgical diseases of the esophagus are treated by gastroenterology surgeons or thoracic surgeons, depending on the country and institution where the patient is being treated, the attending surgeon should be familiar with basic treatment principles and with the interventions that should be considered depending on the individual presentation. It is key to determine the need for acute surgery or for alternative interventions in a timely manner.

Non-operative treatment is appropriate for many patients with iatrogenic perforation (e.g. perforation after dilatation for achalasia or benign strictures). In patients with limited injury to the esophageal wall and contained leakage without systemic symptoms of infection and compromised circulation, careful observation, nil per mouth, appropriate treatment with intravenous broad-spectrum antibiotics and proton pump inhibitors (PPIs) and nutritional support may be sufficient for successful treatment [2]. Non-operative treatment should also be used when the perforation is related to an inoperable malignant stricture. Patient outcome depends mainly on the proper treatment of mediastinal and pleural contamination, and indications for percutaneous drainage or more extensive drainage by surgical intervention should be considered carefully if there is gross contamination.

Primary repair of esophageal perforation is possible, especially in patients admitted to the hospital within 24 hours of the event [6,9,21,35-38]. However, a recent study found that mortality risk was not related to wait time exceeding 24 hours [5]. When repair is attempted in iatrogenic cases with a stricture distal to the perforation, a myotomy might be indicated and the defect covered with a fundoplication. Repair over a T-tube is an alternative treatment that allows for a controlled esophago-cutaneous fistula to be established. This allows healing to take place without contamination [39]. The T-tube can be removed in most patients after 4-6 weeks, and the fistula will eventually close.

Exclusion and diversion of secretions of the esophagus are intended to expedite healing and, at the same time, minimize the risk of further contamination and infection [40]. This approach is rather complex and inconvenient for the patient, and the same results can usually be achieved by simpler procedures.

In the presence of a diseased esophagus with perforation, and in an acute situation, resection of the esophagus may be the most appropriate surgical procedure [1,36,38]. This is a major surgery, and mortality is high (15-40%) and is mainly related to the cause of perforation (caustic perforation in particular), severity of sepsis and the patients' general condition [41]. Reconstruction may be delayed as necessary.

**Figure 4:**
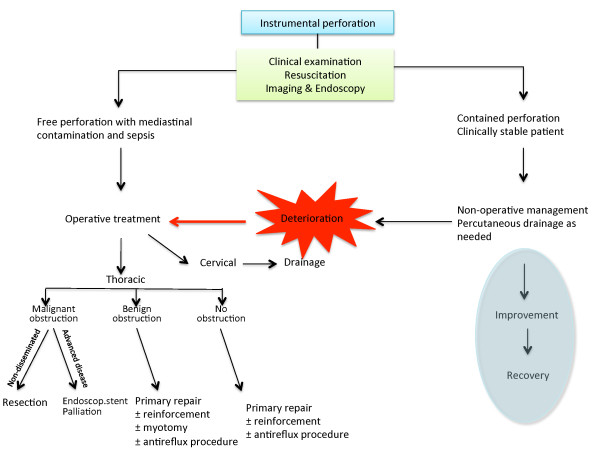
**Management suggestions for *iatrogenic *esophagus perforation [adopted from 41]**.

**Figure 5:**
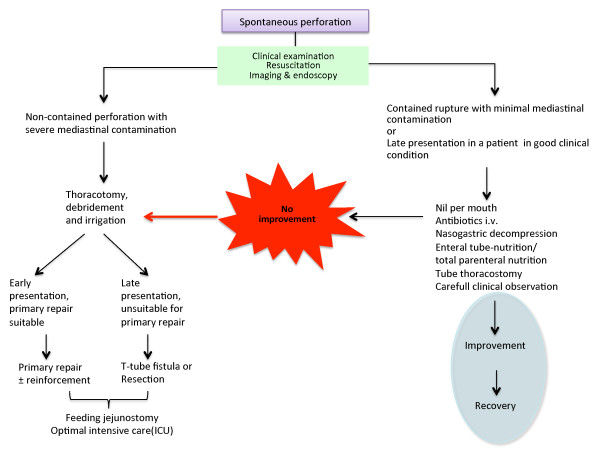
**Management suggestions for *spontaneous *esophagus perforation [adopted from 41]**.

Recent reports emphasize a shift in treatment strategies over the last few decades, with non-operative approaches, such as percutaneous drainage of pleural effusions, collections or abscesses, becoming more common [9,13,42]. In addition, there is a growing use of temporary endoscopic esophageal stents to seal esophageal leakage and to recover gastrointestinal continuity [43-48]. Clinical experience with these treatments is based mostly on smaller series, but in selected patients this treatment seems promising. Use of endoscopic clips for perforation closure has been considered [49], and use of various stent types has been suggested [1,48,50-53]. Endoscopic vacuum sponge therapy has been introduced recently to aid successful drainage and healing of esophageal perforation or anastomotic insufficiency [54,55]. The roles of these new devices in the management of these patients are unclear. Nevertheless, while the rarity of esophageal perforation remains, the toolbox for treatment is growing. Thus, careful evaluation of each patient is mandatory to ensure proper management and care. Although sometimes too simplistic, algorithms can help guide physicians treating perforation of the esophagus. Algorithms for management of iatrogenic and spontaneous esophagus perforations, adopted from suggestions made by Shenfine & Griffin [41], have been expanded and are shown in Figure 4 and 5.

## Discussion

Timely diagnosis and appropriate treatment of esophageal perforation remains challenging, but both are important for managing patients [4,5,9]. Diagnosis can be difficult, due mainly to non-specific symptoms, and should include repeated and extended examination when there is clinical suspicion, even in patients who initially show negative imaging results. There are many treatment options, and a multidisciplinary approach is warranted. A main question would be, whether an immediate operative treatment is indicated or if a less-invasive non-operative approach should be employed. Measures such as antibiotics, PPIs, and so forth can be administered in the ICU as necessary. Percutaneous drainage or endoscopic therapeutic procedures should be considered according to the clinical situation of the patient (Figures 4 and 5).

The very low incidence of this potentially life-threatening condition makes it almost impossible for individual doctors to gain extensive clinical experience. However, referral of patients to a few major hospitals means that there are more of these patients treated at large teaching or university hospitals [4,5,9,56]. Nevertheless, every new case poses a clinical challenge for the physician in charge with regard to clinical evaluation and appropriate diagnostics. Consideration of each patients' overall condition in order to select the most appropriate treatment option adds to treatment complexity and highlights the need for a team approach to support surgical decision-making [9].

The rarity of esophageal perforation make it difficult or impossible to plan for prospective randomized studies with appropriate statistical power. Therefore, current recommendations for management of this group of patients are based mostly on recommendations from smaller institutional or from a few nation-wide patient series.

While there are many treatment options and approaches for perforation of the esophagus, early recognition of suspicious symptoms and signs by the emergency room physician is crucial for prompting the appropriate diagnostic steps. In turn, rapid diagnosis of this often life-threatening condition is critical for expediting the choice of an optimal treatment strategy, whether surgical or non-surgical.

## Competing interests

The authors declare that they have no competing interests.

## Authors' contributions

JAS conceived the study and provided an outline; JAS and AV searched and reviewed the literature, and made the interpretations of available data; JAS and AV drafted and critically revised the manuscript. Both authors read and approved the final manuscript.
